# Application of clinical prediction modeling in pediatric neurosurgery: a case study

**DOI:** 10.1007/s00381-021-05112-z

**Published:** 2021-03-30

**Authors:** Hendrik-Jan Mijderwijk, Thomas Beez, Daniel Hänggi, Daan Nieboer

**Affiliations:** 1 Medical Faculty, Department of Neurosurgery, Heinrich Heine University, Moorenstraße 5, 40225 Düsseldorf, Germany; 2 Department of Public Health, Erasmus University Medical Center, Rotterdam, The Netherlands

**Keywords:** Clinical prediction modeling, Pediatric neurosurgery, Risk assessment

## Abstract

There has been an increasing interest in articles reporting on clinical prediction models in pediatric neurosurgery. Clinical prediction models are mathematical equations that combine patient-related risk factors for the estimation of an individual’s risk of an outcome. If used sensibly, these evidence-based tools may help pediatric neurosurgeons in medical decision-making processes. Furthermore, they may help to communicate anticipated future events of diseases to children and their parents and facilitate shared decision-making accordingly. A basic understanding of this methodology is incumbent when developing or applying a prediction model. This paper addresses this methodology tailored to pediatric neurosurgery. For illustration, we use original pediatric data from our institution to illustrate this methodology with a case study. The developed model is however not externally validated, and clinical impact has not been assessed; therefore, the model cannot be recommended for clinical use in its current form.

## Introduction

Medical doctors increasingly use prediction models to make estimations on patient’s prognosis or diagnosis. Pediatric neurosurgical prediction models are mathematical equations using child-related risk factors—e.g., gender, age, type of hydrocephalus—to calculate the probability of an outcome of interest for that particular child such as cerebrospinal fluid (CSF) diversion revision at 6 months, survival after brain tumor resection, or postoperative cerebellar mutism [1–3].

There has been an increase in the number of articles reporting on prediction models in pediatric neurosurgery [4]. A well-known and widely used prediction model in pediatric neurosurgery is the endoscopic third ventriculostomy success score (EVTSS) [1]. The EVTSS provides an absolute risk estimate for ETV failure at 6 months by means of logistic regression analysis.

Many statistical approaches can be used to develop a prediction model, including but not limited to regression analysis [5]. Detailed statistical output is often presented in the manuscript or in its appendix which is needed to make an adequate evaluation of the presented prediction model.

Herein, the methodology of clinical prediction modeling is presented and illustrated with an original case study. We focus on prediction models developed with logistic regression analysis, although the methodology outlined throughout this article applies to all prediction models.

## Case study

To illustrate the methodology of clinical prediction modeling, we use a set of pediatric patients treated with a ventriculoperitoneal shunt (VP-Shunt) or endoscopic third ventriculocisternostomy (ETV) for hydrocephalus. The data of this set of patients is derived from our own institution and has not been published previously. A simple prediction model including age and gender (Model 1) is compared with a more complex model adding the neurosurgical technique for CSF diversion to the simple model (Model 2).

## Methodology

**Fig. 1:**
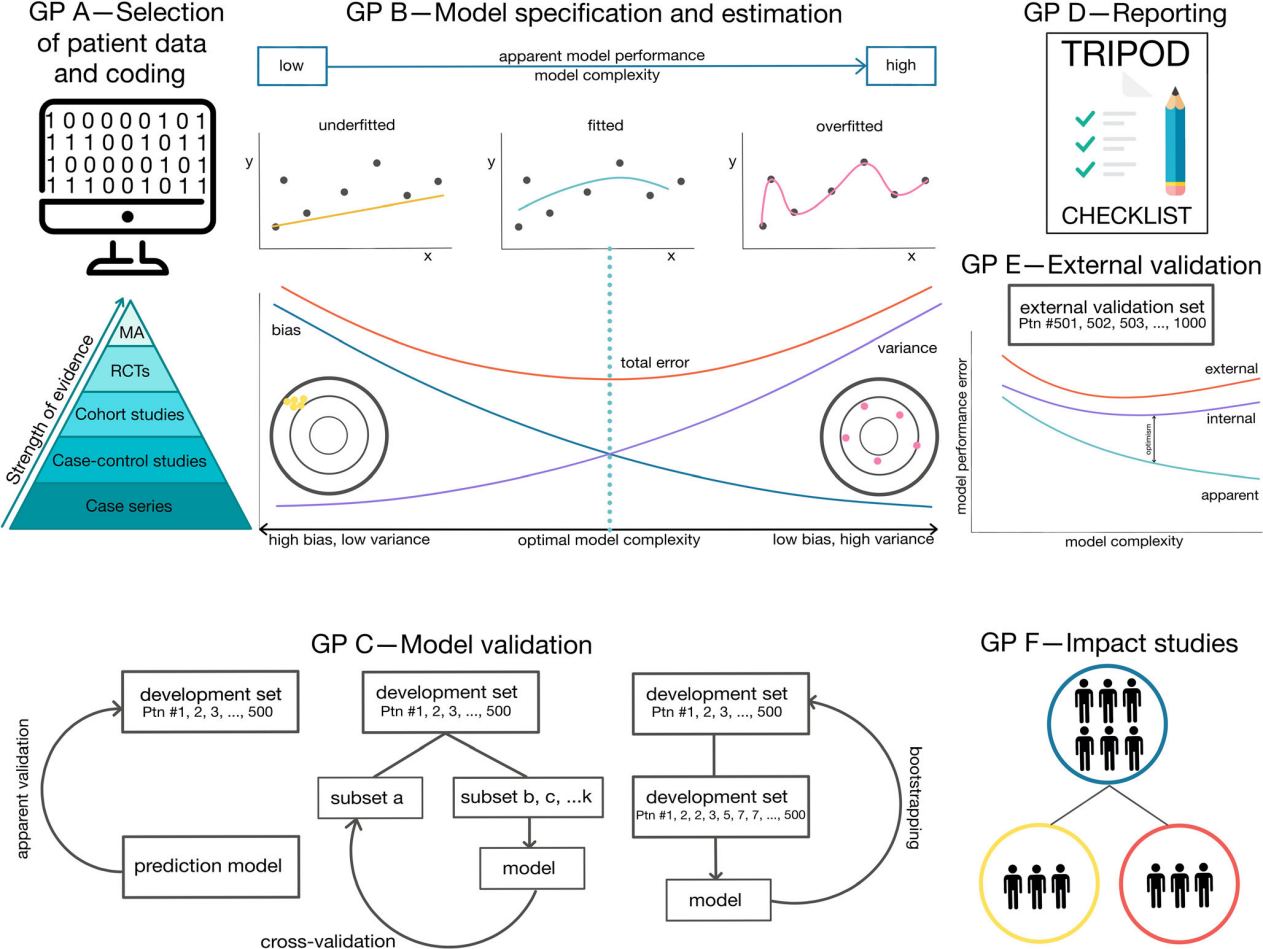
Guideposts (GP) for several steps for the development of clinical prediction models in pediatric neurosurgery. The process of clinical prediction consists of three phases: model development (GP A–D), model validation and/or updating (GP E), and model evaluation by impact studies (GP F). GP A: It is best to select the candidate prognostic variables by subject matter knowledge and thorough literature review. Data curation including coding of variables should be done rigorously. GP B: Be aware of the risk of overfitted prediction models. GP C: At cross-validation, the model development set is divided into subsets. For example, subset *a* functions as a validation set. In the other subsets (*b*, *c*, …, *k*) the model is refitted with *a* as validation set. This process is repeated until each of the subsets has served as a validation set. GP D: Adherence to the TRIPOD checklist is recommended, which can be downloaded from https://www.tripod-statement.org/. GP E: Model performance normally decreases at internal and external validation. GP F: Impact studies are considered imperative for clinical uptake. Part of the contents of this figure is based on previous literature reporting on clinical prediction models [5–7]

The glossary in Box 1 explains some of the terminology often used in prediction modeling. For the development of prediction models with regression analysis, it is advised to systematically follow distinct steps (Fig. 1) [5]. However, prior to the start of the development of a new prediction model, existing models should be searched for [5]. It is recommended to validate and/or update existing models instead of developing de novo prediction models.

Box 1 Glossary for common used terminology in clinical prediction modeling

**Table:** 

*Apparent model performance—*Performance of the model in the patient sample used for ist derivation.	
*Bias-variance trade-off/total error—*The resultant of error due to bias and error due to variance. It is a trade off, because it is impossible to reduce both.	
*Bias—*The ability of the model to capture the data. It is the systematic difference between estimated parameters and the true parameters.	
*Calibration—*Model performance measure that shows the agreement between the predictions of the model with the observed outcomes. Good calibration is essential when using the prediction model for clinical decision making.	
*Discrimination—*Ability of a prediction model to discriminate between patients with the event of interest and without the event of interest. Often quantified using the *c*-statistic.	
*EPV—*Events per variable: the ratio between the number of outcomes of interest and the number of degrees of freedom of prognostic variables. The number of events is the smaller of the number of patients having the event or not having the event.	
*External validity—*The generalizability (or transportability) of the model to similar but different patients.	
*Internal validity—*The reproducibility check of the developed model by assessing optimism in model performance.	
*Optimism—*True model performance minus apparent model performance.	
*Overfitted model—*Overly complex prediction model that does not generalize well on new sets of patients due to fitting data idiosyncrasies in the data set used for its derivation.	
*Stepwise selection procedure—*Data-based inclusion of prognostic variables into the prediction model based on statistical thresholds.	
*Subject matter knowledge—*Inclusion of prognostic variables into the prediction model is based on expert opinion and/or thorough review of the literature.	
*TRIPOD*—Guideline to be used for Transparent Reporting of a Multivariable Prediction Model for Individual Prognosis or Diagnosis. Available at https://www.tripod-statement.org/.	
*True model performance—*Performance of the model in the underlying/source population.	
*Variance—*The statistical uncertainty in the estimated parameters. Prediction models with high variance can provide widely different predictions if re-estimated on new data sets.	

### Description of patient data and data preparation

The objective of the model should be clearly defined at the start, e.g., what is the clinical decision the model needs to support. Next, a description on how patients were selected for inclusion in the prediction analysis is imperative, because data are often primarily collected for other purposes and different study designs have different consequences on the interpretation of the results [5]. For example, for a diagnostic prediction model, a cross-sectional cohort study design is preferred, whereas for a prognostic prediction model, a longitudinal prospective cohort study design is preferred. Retrospective cohort study designs are most often used due to its ease in data collection and time effectiveness. The results derived from retrospective study designs may be however hampered by selection bias. Data from randomized controlled trials are normally of high quality but the (potential lack of) generalizability to other patients may delay clinical uptake of the model. The patient sample should be as representative as possible for the intended population.

Selection of candidate prognostic variables is ideally done before the start of the study. *Subject matter knowledge* and adequate review of the literature are incumbent to get the ultimate set of prognostic variables (Fig. 1).

Data sets are rarely complete. In general, there are two options to deal with missing data. First, a complete case-analysis can be performed. Patients with missing data are excluded. This may reduce the sample size in a way that a valid prediction model cannot be generated. Furthermore, the reason for missing data is likely not completely at random and the results obtained by the prediction model may be biased accordingly. A better strategy is to replace the missing entries with reliable values if missingness is substantial, i.e., more than 5%[8], to maintain enough statistical power for model development. Multiple imputation is then often the most sensible method. The available data of the patient and comparable patients are used to estimate the missing value. This method should be used sensibly as it makes assumptions on the data and mechanisms of the occurrence of missing data. More details on multiple imputation can be found elsewhere [9].

It is vital to ensure a robust sample size relative to the model complexity. A model can be made overly complex by considering too many prognostic variables (Fig. 1) [5]. Complex models are at high risk of overfitting: the results look promising but do not generalize to new sets of patients (Fig. 1). To limit overfitting, traditionally, 10 events per prognostic variable (*EPV*) as a minimum are suggested. Thus, not the total sample size but the number of events is the effective sample size in the of field of prediction modeling.

EPV is a widely used term; however, it should be noted that the number of variables considered is the total number of estimated parameters of the considered variables. As a result, considering surgical resection including three categories (total resection, subtotal resection, and biopsy) as prognostic variable requires two parameters to be tested and needs therefore a larger effective sample size. The EPV ratio is an easy rule-of-thumb to determine the total sample size required to develop a prediction model; however, recently, several approaches have been suggested to base the sample size calculations on the expected degree of overfitting, taking into account the number of parameters to be estimated and expected variation to be explained as this can substantially impact the required sample size beyond the EPV [10–12].

Adequate coding of prognostic variables is an important part of data preparation (Fig. 1). It is not recommended to dichotomize or categorize continuous variables such as age to prevent loss of prognostic information [13].

#### Case study

The case study involves a retrospective analysis of 63 hydrocephalic children admitted to our tertiary center for a CSF diversion procedure. The study focused on sociodemographic data including age and patient gender, and on the applied neurosurgical technique. Ethical approval was obtained from our local institutional review board (IT-TEMP 50). Fourteen children needed a revision of the initial procedure at 6 months. Thus, the number of predictors relative to the number of events (14 revision procedures) was larger for Model 2. The variables—identified a priori—were all easy to collect. A descriptive of the patient characteristics can be found in Table 1.

**Table 1:** Description of patient characteristics

	Revision at 6 months	
	No (*n* = 49)	Yes (*n* = 14)
Gender		
Male (*n*, %)	33 (86.8%)	5 (13.2%)
Female (*n*, %)	16 (64%))	9 (36%)
Age (mean, SD)	7.1 (6.8)	5.6 (6.4)
CSF diversion		
VP-Shunt (*n*, %)	25 (80.6%)	6 (19.4%)
ETV (*n*, %)	24 (75.0%)	8 (25%)

*CSF* cerebrospinal fluid, *ETV* endoscopic third ventriculocisternostomy, *VP-Shunt* ventriculoperitoneal shunt

### Model specification and estimation

Clinical prediction models should be as simple as possible to facilitate clinical uptake by neurosurgeons. However, if models are too simplistic, the performance will be limited, hampering clinical uptake by neurosurgeons. The ultimate goal of prediction models is to give valid predictions in new patients. The expected prediction error of a model can be decomposed in a *bias* term and *variance* term. Relatively simple models are expected to have high bias, but low variance—they are at risk of underfitting the data. Complex models (e.g., by inclusion of too many predictors) have low bias, but high variance—they are at risk of overfitting the data. The challenge in developing a prediction model is to balance bias and variance to ensure good performance in new patients. The least total error, i.e., the combination of the bias and variance, is normally found in a fitted model (Fig. 1).

There are several options to select a combination of prognostic variables for the prediction model. Ideally, the set of candidate prognostic variables is defined before the start of the study by means of subject matter knowledge or thorough literature review. If many candidate prognostic variables are of interest, data-driven prognostic variable selection is often applied to reduce the number of candidate prognostic variables. However, these statistical strategies have many drawbacks, especially when applied to small data sets. Automated *stepwise selection procedures*, mostly backward elimination, tend to provide too extreme predictor effects due to repeated significance testing. Another way of statistical variable selection is to univariably test for significance in the prognostic variable—outcome association and then include the most prominent associations into the prediction model. However, the strength of a prognostic variable also depends on its distribution in the data set used for model generation. Thus, a rare prognostic variable having a strong association with the outcome will likely have less prognostic potential compared with a common prognostic variable with a less strong association with the outcome. There are more data-driven strategies of selecting relevant prognostic variables. These all suffer from risk of overfitting due to repeated significance testing. A more liberal *p*-value, for instance *p* < 0.20, for variable selection may help limiting the risk of overfitting.

In the next step, the parameters (i.e., the regression coefficient of the prognostic variables and the model intercept) of the model are estimated. It is common to use linear regression, logistic regression, and Cox survival analysis for continuous, categorical, and time-to-event outcomes respectively (Box 2) [5]. These models are based on assumptions such as the additivity assumption and proportionality assumption (Box 2). Modifications of the statistical model to address model assumptions and predictor-outcome relations may result in a well-fitting model, with high *apparent model performance* in the development set used for its generation (Fig. 1). Yet, the model has become more complex and may not generalize well to other sets of patients—i.e., overfitting (Fig. 1). According to the effective sample size, sample sizes with a low number of events are therefore at higher risk. Statistical shrinkage techniques aim to limit overfitting. These methods shrink the coefficients of the prognostic variables [14]. Uniform shrinkage techniques reveal a shrinkage factor (determined by a heuristic formula or a bootstrapping procedure) that should be applied to the regression coefficients after the estimation procedure. Regularized regression methods use statistical shrinkage techniques such as penalized maximum likelihood estimation and the least absolute shrinkage and selection operator (LASSO) to limit the risk of overfitting during the model estimation procedure, although these methods are of limited use as sample sizes become very small [15]. The best method is however to minimize the use of statistical testing by using subject matter knowledge [16].

Box 2 Risk prediction with statistical regression methods

**Table:** 

The type of the outcome of interest determines the regression method that is used. *Continuous outcomes: linear regression* If the outcome is assessed on a large numerical scale, then the outcome is likely continuous. Patient-reported outcome measures including quality of life questionnaire are typically evaluated by linear regression. For an individual patient, risk prediction may come from *y* = *α* + *β*_1_*x*_1_ + *β*_2_*x*_2_ + ⋯ + *β*_*k*_*x*_*k*_ Here, *y* is the outcome of interest. The parameters of this regression model include the *α* (intercept) and *β* (regression coefficient). The prognostic variables are denoted by the *x*, and are weighted by their corresponding regression coefficient (*β*). Well-known model assumption: additivity of effects on the outcome. *Categorical outcomes: logistic regression* If a binary (i.e. “yes” or “no”) outcome, for example, revision of a VP-Shunt, is truly known for all children at a particular time point, absolute risk prediction can be calculated from a transformation of the binary logistic regression function: $p=\frac{\exp \left(\alpha +{\beta}_1{x}_1+{\beta}_2{x}_2+\cdots +{\beta}_k{x}_k\right)}{1+\exp \left(\alpha +{\beta}_1{x}_1+{\beta}_2{x}_2+\cdots +{\beta}_k{x}_k\right)}$ Here, *p* denotes the probability of having the outcome. It should be noted that the probability given by a logistic regression formula is always wrong. A probability ranges from 0 to 100%. A patient, however, will either experience the outcome of interest (100%) or not (0%). If an outcome has multiple categories, extensions of the logistic regression formula are available. Well-known model assumption: multiplicative effect on the odds of the outcome. *Time-to-event outcomes: survival regression* If the outcome of interest is time until an event occurs, Cox survival regression is usually applied. These models consider the time between a starting point such as surgical resection of a brain tumor until death or another endpoint. Patients that are lost to follow-up are censored, making this analysis unique. To predict survival the survival probability of an individual patient (that is, the patient has not experienced the outcome), a transformation of the Cox model—the survival function *S*(*t*)—is normally used: $S(t)={S}_0{(t)}^{e^{\left({\beta}_1{x}_1+{\beta}_2{x}_2+\cdots +{\beta}_k{x}_k\right)}}$ *S*_0_(*t*) represents the baseline hazard. It is vital that the baseline hazard at a particular time point is always presented in the article. Well-known model assumption: proportionality of the hazard ratios.	

#### Case study

Binary logistic regression was used to estimate the model parameters (Table 2). No statistical variable selection procedure was applied. Bootstrapping was used to determine the shrinkage factor. For the 2-predictor model, we found a shrinkage factor of 0.87. A shrinkage factor of at least 0.90 is typically aimed for when planning clinical prediction models [11, 12]. For the 3-predictor model, the shrinkage factor is 0.71. This indicates that the latter model suffers from more overfitting. Thus, the regression coefficients should be multiplied by 0.87 and 0.71, respectively, to obtain more reliable predictions in other pediatric patients. We note that shrinkage methods for prediction models should be applied sensibly because it may not be a solution to every data set [17].

**Table 2:** Multivariable prediction models for revision of CSF diversion at 6 months

	Model 1	Model 2
Gender (*β*; OR, 95% CI)	1.42; 4.1 (1.1, 14.8)	1.51; 4.5 (1.2–16.7)
Age (*β*; OR, 95% CI)	−0.05; 0.95 (0.86, 1.05)	−0.08; 0.92 (0.82–1.03)
Neurosurgical intervention (*β*; OR, 95% CI)		0.75; 2.1 (0.5, 8.2)
Intercept	−1.60	**−**1.90
Shrinkage factor (slope)	0.87	0.71
*c*-statistic	0.71	0.73
Optimism-corrected *c*-statistic	0.67	0.66

*β* regression coefficient, *CI* confidence interval, *CSF* cerebrospinal fluid, *OR* odds ratio

The full regression equation for Model 1:

$$ \log \frac{p}{1-p}=\mathrm{logit}\kern0.20em (p)=-1.60+\left(1.42\times \mathrm{Gender}=\mathrm{female}\right)+\left(-0.05\times \mathrm{Age}\right) $$

The full regression equation for Model 2:

$$ \log \frac{p}{1-p}=\mathrm{logit}\kern0.20em (p)=-1.90+\left(1.51\times \mathrm{Gender}=\mathrm{female}\right)+\left(-0.08\times \mathrm{Age}\right)+\left(0.75\times \mathrm{Neurosurgical}\kern0.34em \mathrm{intervention}=\mathrm{ETV}\right) $$

### Model performance

For clinical uptake it is vital that the prediction model discriminates well between children having the event and children not having the event. The potential to discriminate is given by the *c*-statistic. The *c*-statistic calculates the probability that the prediction model provides a higher score for a randomly selected child with the outcome compared with a randomly selected child without the outcome [5]. A *c*-statistic < 0.50 means that model is worse than guessing, a *c*-statistic of 0.50 means that the model has no discriminative ability, and a *c*-statistic of >0.50 shows that the model has predictive potential. A *c*-statistic of 1.0 represents perfect *discrimination* by the model. For a binary outcome, the *c*-statistic is equivalent to the area under the curve of the receiver operating characteristic curve. Despite good discriminative ability, the prediction model might systematically overestimate or underestimate the risk of a child.

**Fig. 2:**
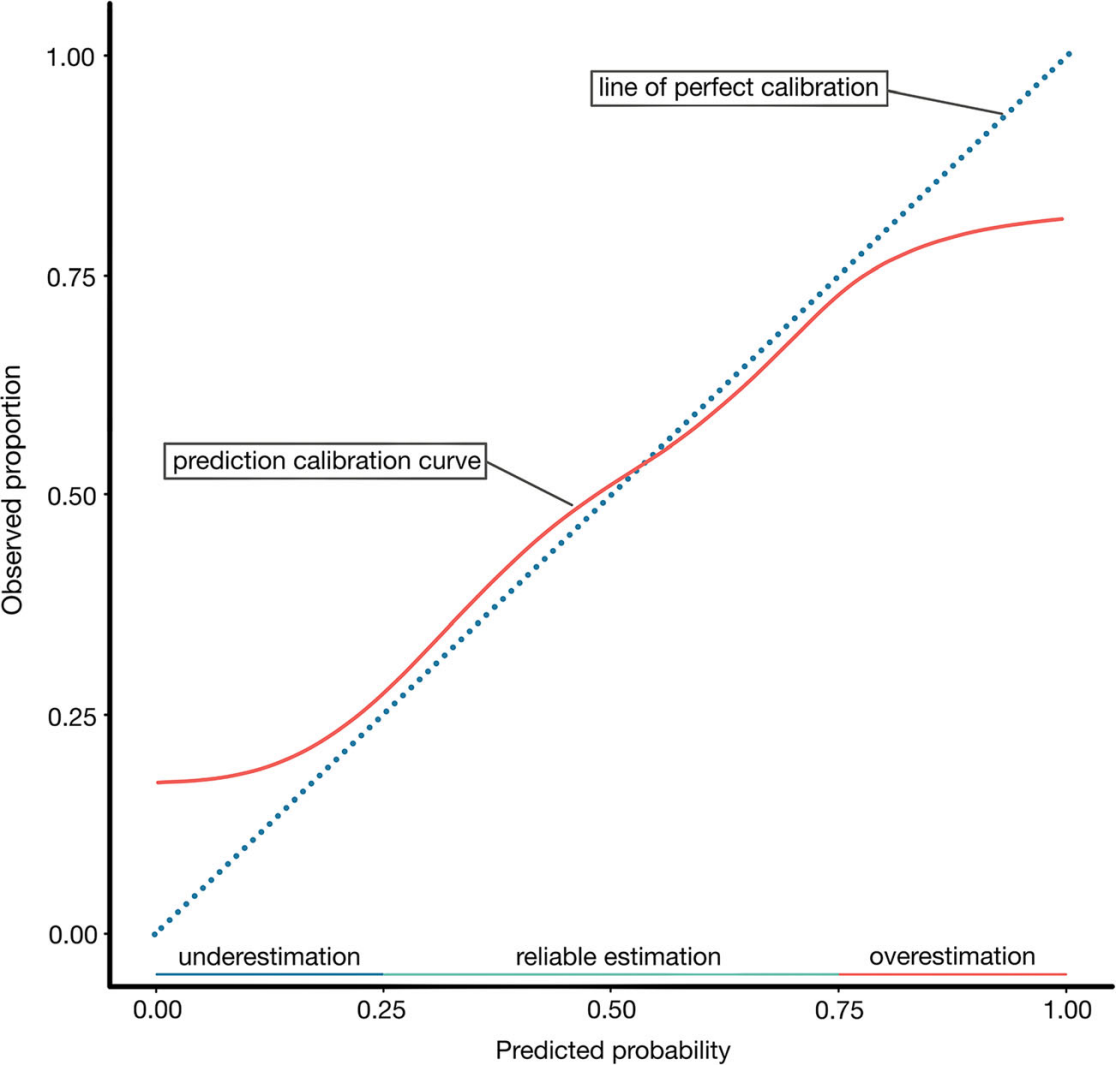
Calibration plot for a typical overfitted prediction model: overestimated risks for high-risk patients and underestimated risks for low-risk patients. If such a model calculates a risk of 90% and thereby identifies a child as high-risk, the pediatric neurosurgeon should taken this caveat into account when applying this model in clinical practice. Calibration is the Achilles heel of predictive analytics [18]

*Calibration* methods gauge the accuracy of the model. It is vital for a prediction model that the predicted probabilities by the model are in line with the observed outcomes. This agreement is illustrated in a calibration plot (Fig. 2). The diagonal represents perfect calibration. Ideally, the calibration plot corresponds to the diagonal, suggesting perfect calibration which occurs in utopia only [19]. An *overfitted model* typically underestimates low-risk patients and produces overestimated results in high-risk patients (Fig. 2).

Even with acceptable discrimination and calibration, clinical utility is not always guaranteed. Decision curve analysis investigates the potential clinical usefulness of a prediction model [20]. The clinical usefulness of a prediction model is then quantified in terms of the net benefit across a range of clinically relevant decision thresholds (thresholds at which a neurosurgeon would treat high-risk patients and not treat low-risk patients) compared with default strategies of treating all patients or none of the patients. If the net benefit of the model is higher compared with default strategies, then the model is suggested to be clinically useful. A more detailed explanation is beyond the scope of this article, and we refer to more specified literature [21, 22].

Overall model performance measures are sometimes given; these measure the overall performance of a prediction model and hence are a combination of the model’s discriminative ability and calibration. For example, the explained variance (*R*^2^) index ranges from 0 to 100%. *R*^2^ assesses the proportion of the variability in the outcome that is explained by the prediction model, which is typically below 50%. Other measures of overall model performance are pseudo *R*^2^ values for logistic regression and survival analysis or the Brier score.

#### Case study

As expected, we observed an increasing *c*-statistic with increasing model complexity: Model 1 yielded a *c*-statistic of 0.71, whereas Model 2 had a *c*-statistic of 0.73 [23]. Calibration plots are normally evaluated at external validation attempts. By lack of an independent validation set, calibration was not considered.

### Model validation

Model validation is a crucial aspect of generating robust prediction models (Fig. 1). Developed prediction models tend to have too optimistic apparent model performance measures when applied to the data used for development. Therefore, it is a prerequisite for every single prediction model to proceed with an *internal validation* procedure (Fig. 1). Internal validation techniques aim to estimate the model performance when applying the model to similar patients not used for model development and quantify optimism between the model performance measures accordingly. These techniques use the same data that were used for model development. A commonly used method, yet inefficient method as it decreases the sample size, is the split sample approach. Here the development set is randomly split into a development and a validation set. In addition, the validation set only differs by chance from the development set. Therefore, it is highly recommended to perform more efficient resampling methods: bootstrapping or cross-validation. A bootstrap sample is created using random sampling of patients with replacement from the development data set to mimic random sampling from the source population of the patients. Thus, patients can be selected several times in one bootstrap sample (Fig. 1). Bootstrap samples are of the same size as the development data set and are repeatedly drawn to reveal a large number, for example, 1000, of bootstrap data sets. On each of the bootstrapped data sets, all model development steps are repeated (including variable selection). The difference in model performance of these developed models when evaluated on the bootstrapped and original patient set is called the *optimism* (Fig. 1). An alternative technique that can be used is cross-validation. Here, all the data is divided into subsamples. One of these subsamples is used for validation, and the other is used for development. This procedure is then repeated several times, e.g., 10 times in a 10-fold cross-validation. To assess the performance measures of the model, the results from all test sets are used. A stronger version of cross-validation is internal-external validation. Here a non-random split of the data is used to split the data in different subsamples, e.g. center or country.

*External validation* addresses the generalizability (or transportability) of the model to similar but different patients (Fig. 1). In contrast to internal validation, external validation is able to address the heterogeneity in the patients of the population of interest in real-life. Ideally, the outcome and prognostic variables of interest are easily to collect and assessed without measurement error. At external validation, the steps for model developed are not repeated, nor is the model refitted in the external data set. However, the developed model with its parameters is applied to the new external set of similar patients and the model performance measures are quantified accordingly. Consequently, the generalizability of the model can be judged. There are three types of external validation: temporal, geographical, and domain validation. In temporal external validation, the external set of patients comes from the same institution but in different time period. In geographical external validation, the new set of patients comes from different institutions or countries. In domain validation, the model is tested on patients very different from the development patient set. For example, a model has been developed in adults and validated in children. For a reliable external validation study, at least 100 events have been considered as minimum [24], although recent research proposes a more tailored sample size approach [25].

#### Case study

The two models were internally validated using 1000 bootstrap samples. The optimism was calculated: The discriminative performance of the 2-predictor model dropped from 0.71 to 0.67. The drop in *c*-statistic was larger for the 3-predictor model: from 0.73 to 0.66. An independent set of patients was not available, and therefore, the model could not be subjected to external validation. Therefore, this model is immature in its current form and cannot be recommended for clinical use accordingly.

## Results

Reporting results from prediction models accurately is crucial for future work (Fig. 1). Without the full prediction model including all the parameters (model intercept and regression coefficients), colleagues are unable to use the model properly. The quality of reporting has generally been poor. Therefore, in 2015 and 2020, the Transparent Reporting of Multivariable Prediction Model for Individual Prognosis or Diagnosis (*TRIPOD*) and TRIPOD for Abstracts are published [26, 27]. These guidelines include a checklist authors should ideally follow when reporting their results.

To aid clinical use, prediction models can be presented in many ways such as score charts and nomograms—however, again, the full regression equation should always be presented. If a simplified version of a model is deemed necessary for presentation, then this new model should be validated as well to evaluate its performance with respect to the full model [28]. Nowadays, it is relatively easy to create a user-friendly web-based instrument or nomogram to visualize and calculate the individual probability of a patient.

### Case study

The full prediction model can be derived from Table 2. For prognostication, the risk score for a 7-year-old boy undergoing an ETV procedure for hydrocephalus is calculated by the following formula:

$$ \mathrm{Risk}\kern0.34em \mathrm{score}=-1.90\kern0.20em \left(\mathrm{intercept}\right)+\left(1.51\ast 0\kern0.20em \left(\mathrm{male}\kern0.34em \mathrm{gender}\right)\right)+\left(-0.08\ast 7\kern0.20em \left(\mathrm{age}\kern0.20em \mathrm{in}\kern0.34em \mathrm{years}\right)\right)+\left(0.75\ast 1\left(\mathrm{ETV}\right)\right)=-\mathrm{1.71.} $$

Consequently, the probability of a 6-month CSF diversion revision for this child equals:

$$ \exp \left(-1.71\right)/\Big(1+\exp \left(-1.71\right)=15\%. $$

## Discussion

In this paper, the fundamentals of developing a clinical prediction model are described and illustrated with an original case study. We focused primarily on the model development stage.

As demonstrated by the case study, overfitting is an important pitfall to consider when developing a clinical prediction model. Either the use of a development data set with a low number of events or the use of multiple candidate prognostic variables make a clinical prediction model prone for overfitting. Prior to uptake in clinical practice (despite a sensible modeling strategy), a prediction model should ideally follow three stages: model development, model validation and/or updating, and model evaluation by impact studies (Fig. 1).

Updating a clinical prediction model can help to improve a poorly performing model at external validation [29, 30]. The information from the developed model is combined with the patients from the external validation set. For example, if the event rate of revision of a CSF diversion procedure is lower in an external validation set, the predicted risks by the model of the presented case study may be overestimated. Simply adjusting one parameter of the model—i.e., the intercept—may then be enough to get the model tailored to the local circumstances of the new set of patients. Further, variations in case mix, new promising biomarkers, or other innovations may cause calibration drift of the initial developed prediction model causing flawed predictions [31]. To re-balance the equation, it is recommended to update clinical prediction models regularly. Methods for updating include but are not limited to modification of baseline risk, modification of the regression coefficients, and model extension with new predictors [31–34]. Using updating techniques, the performance of the prediction model likely increases and prevents the development of multiple de novo prediction models.

The last stage includes the implementation of the clinical prediction model with evaluation of its clinical impact (Fig. 1). The need for a comparative study design—ideally randomized trials—makes this step unique, albeit difficult to conduct [33]. An intermediate step to clinical implication of a prediction model can be the application of decision analytic techniques. These techniques, including net benefit approaches and decision curves, evaluate the proposed model against the current standard of care [20–22].

Prediction models cannot take over the decision-making process of the pediatric neurosurgeon [35]. No prediction model can activate an individual treatment plan. However, these models aim to help in the decision-making process, especially for clinical conditions in equipoise regarding optimal patient management. Prediction models may also help to communicate anticipated future events of diseases to children and their parents. This may enhance the patient–doctor relation accordingly. Therefore, pediatric neurosurgeons should ideally have a basic knowledge on how to interpret and use clinical prediction models. Clearly, prediction models may not be always readily understandable by pediatric neurosurgeons. Therefore, to ensure safe use of prediction models in clinical practice, regulatory standards for prediction models have been proposed recently [36]. For the interested reader, other explanatory literature provides further and more detailed information [14, 26, 35, 37–40].

We are aware that other approaches to model development are available, such as machine learning techniques. Where statistical regression techniques rely more on subject matter knowledge and use prespecified mathematical algorithms, machine learning techniques are more data driven relying on highly flexible self-learning automatized algorithms [41]. Consequently, opaque models and black boxes may emerge jeopardizing the interpretability of the results. Machine learning techniques often do not perform better than statistical regression techniques for predicting outcomes with limited sample sizes [42]. However, machine learning techniques have shown great promise in for example imaging interpretation [43]. It is still believed that regression methods will remain a default framework for clinical prediction modeling.

### Case study

The case study presented here serves as an illustration. External validation of the score as well as evaluation of its clinical impact has not been performed. Therefore, this example prediction model for revision of a CSF diversion procedure at 6 months is clearly premature and cannot be recommended for use in clinical practice.

To conclude, the process of generating sensible clinical prediction models warrants a systematic approach with a multidisciplinary team including experts in the medical, epidemiological, and statistical field since multiple aspects are to be considered. Therefore, adherence to relevant guidelines is highly recommended.
